# Comparative Transcriptomic Analysis Revealing the Potential Mechanisms of Erythritol-Caused Mortality and Oviposition Inhibition in *Drosophila melanogaster*

**DOI:** 10.3390/ijms25073738

**Published:** 2024-03-27

**Authors:** Lei Li, Hongrui Duo, Xiaoxi Zhang, Huiming Gong, Bo Li, Youjin Hao

**Affiliations:** College of Life Science, Chongqing Normal University, Chongqing 401331, China; lilei139334@gmail.com (L.L.);

**Keywords:** *Drosophila melanogaster*, erythritol, RNA-Seq, osmolality, mortality mechanism

## Abstract

Erythritol has shown excellent insecticidal performance against a wide range of insect species, but the molecular mechanism by which it causes insect mortality and sterility is not fully understood. The mortality and sterility of *Drosophila melanogaster* were assessed after feeding with 1M erythritol for 72 h and 96 h, and gene expression profiles were further compared through RNA sequencing. Enrichment analysis of GO and KEGG revealed that expressions of the adipokinetic hormone gene (*Akh*), amylase gene (*Amyrel*), α-glucosidase gene (*Mal-B1/2*, *Mal-A1-4*, *Mal-A7/8*), and triglyceride lipase gene (*Bmm*) were significantly up-regulated, while insulin-like peptide genes (*Dilp2*, *Dilp3* and *Dilp5*) were dramatically down-regulated. Seventeen genes associated with eggshell assembly, including *Dec-1* (down 315-fold), *Vm26Ab* (down 2014-fold) and *Vm34Ca* (down 6034-fold), were significantly down-regulated or even showed no expression. However, there were no significant differences in the expression of three diuretic hormone genes (*DH44*, *DH31*, *CAPA*) and eight aquaporin genes (*Drip*, *Big brain*, *AQP*, *Eglp1*, *Eglp2*, *Eglp3*, *Eglp4* and *Prip*) involved in osmolality regulation (all *p* value > 0.05). We concluded that erythritol, a competitive inhibitor of α-glucosidase, severely reduced substrates and enzyme binding, inhibiting effective carbohydrate hydrolysis in the midgut and eventually causing death due to energy deprivation. It was clear that *Drosophila melanogaster* did not die from the osmolality of the hemolymph. Our findings elucidate the molecular mechanism underlying the mortality and sterility in *Drosophila melanogaster* induced by erythritol feeding. It also provides an important theoretical basis for the application of erythritol as an environmentally friendly pesticide.

## 1. Introduction

Pesticides play a crucial role in agricultural production by minimizing losses and enhancing crop yield and quality [1]. While synthetic pesticides currently dominate the market, only a mere 1% effectively targets pests on the intended plants [2]. The excessive use of these chemicals results in a significant amount of residuals spreading to non-target plants and environmental surroundings, eventually entering the food chain and posing risks to human health [3,4]. This underscores the urgent global need for environmentally friendly and efficient alternatives.

Polyol sweeteners have recently attracted significant interest as pesticides. Erythritol is a four-carbon sugar alcohol produced by a variety of plants, fungi and microbes [5]. It has been shown to be toxic to a wide variety of insects, including the fruit fly, termite, house fly, stable fly, pear psylla, mosquito, and ant [6,7,8,9,10,11]. *Drosophila melanogaster* shows a dietary preference for erythritol, which not only causes death in a concentration-dependent pattern but also impairs their motility [12]. Donnell et al. reported that erythritol dramatically suppresses the egg production of female *D. melanogaster*, arrests larval development, prevents them from reaching the pupa stage and eventually kills them [13]. Similarly, erythritol was effective in reducing the survival and fecundity of *Drosophila suzukii* as well as affecting its physiological excretion [14]. Erythritol remarkably decreased the survival of ants (*Solenopsis invicta* Buren, *Tetramorium immigrans* Santschi, *Formica glacialis* Wheeler, *Camponotus subarbatus* Emery, and *Camponotus chromaiodes* Bolton) in a concentration-dependent manner [11]. Interestingly, worker ants bring erythritol to the colony and feed it to their members at toxic doses, resulting in their death. Furthermore, erythritol could significantly impair *D. suzukii* motility as well as the walking frequency and flight ability of *Bactrocera dorsalis* [15].

Although some studies have indicated that erythritol ingestion can induce insect mortality and affect their reproductive capabilities, the precise molecular mechanisms underlying these phenomena remain unresolved. Several hypotheses regarding the molecular mechanisms have been proposed: (1) erythritol remains unhydrolyzed in the midgut but accumulates in the hemolymph, resulting in elevated osmolality and eventual mortality [16]; (2) erythritol impedes the absorption of other nutrients in the midgut, causing starvation and death [12]; (3) erythritol decelerates food evacuation, inducing a sense of fullness in insects and reducing feeding, ultimately resulting in death by starvation [17]; (4) ingestion of erythritol triggers excessive reflux, resulting in dehydration and eventual demise [17]; and (5) erythritol induces alterations in the microbial community within the midgut [18].

In this study, transcriptome sequencing was performed on *D. melanogaster* (henceforth referred to as the fly) fed a normal medium and erythritol medium. Through comparative transcriptomic analysis, we found that the energy metabolic pathway was up-regulated and genes related to egg synthesis were severely down-regulated in erythritol-fed flies. Our findings elucidated that the cause of fly mortality was primarily attributed to energy deficiency rather than a significant alteration in osmolality. Moreover, the energy deficiency compelled the flies to allocate their limited energy towards basic survival rather than reproductive processes.

## 2. Results

### 2.1. Effect of Erythritol on Fly Survival and Egg Production

**Figure 1:**
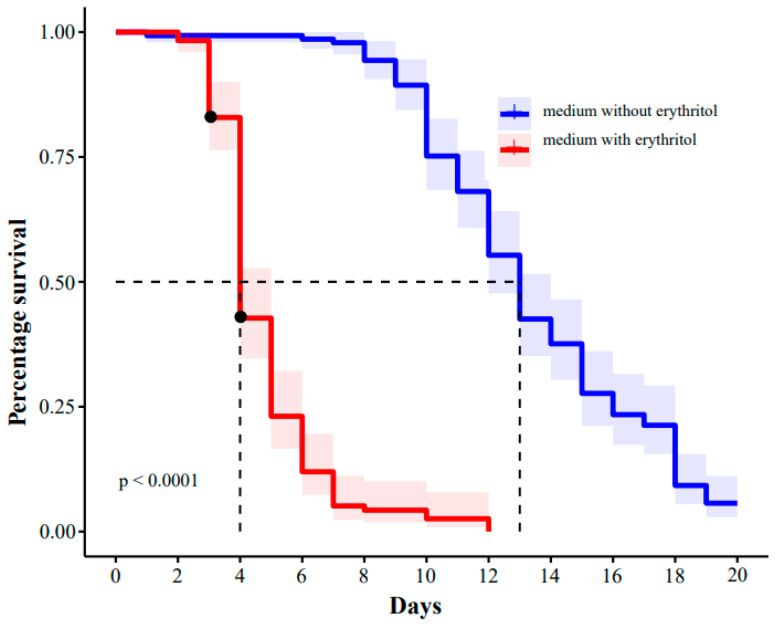
Survival curve of flies fed media with or without erythritol. Erythritol-fed fruit flies reached 20% mortality on day three and 60% mortality on day four. Control fruit flies reached 20% mortality on the tenth day and 60% mortality on the thirteenth day.

The survival rate of flies fed with erythritol was significantly shorter than those in the control group (Figure 1). Flies fed erythritol medium started to die on day two and had a mortality rate of about 60% on day four, while flies fed normal medium had a mortality rate of 60% on day fourteen. In addition, flies fed erythritol medium were significantly less motile than flies fed normal medium and were unable to lay eggs, indicating that erythritol significantly impaired the survival and egg production.

### 2.2. Effect of Erythritol on Gene Expression

**Figure 2:**
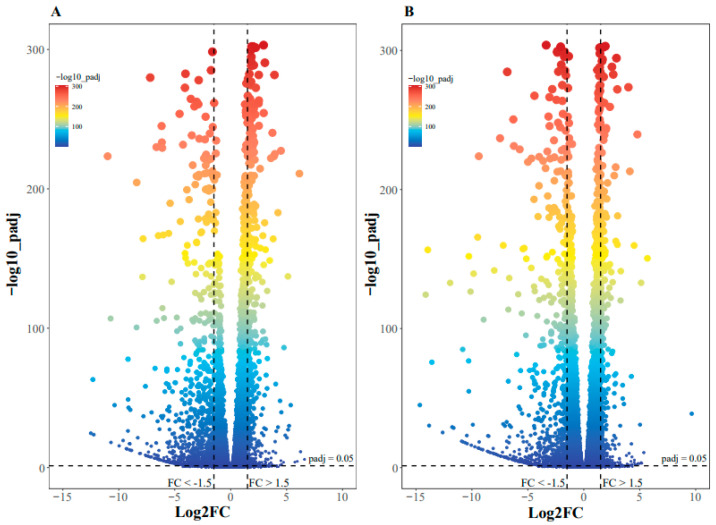
(**A**) Differentially expressed genes between erythritol-treated flies and control flies after 72 h based on a log2fold change >1.5 or <-1.5, and padj < 0.05. (**B**) Differentially expressed genes between erythritol-treated flies and control flies after 96 h based on a log2fold change >1.5 or <-1.5, and padj < 0.05.

Erythritol-fed flies showed noticeably altered gene expression patterns. As shown in Figure 2, for T72h (flies treated with erythritol for 72 h) vs. CK72h (CK72h: flies treated without erythritol for 72 h), 1276 genes were down-regulated, while 581 genes were up-regulated. As for T96h (flies treated with erythritol for 96 h) vs. CK96h (flies treated without erythritol for 96 h), 1020 genes were down-regulated, whereas 599 genes were up-regulated. It is well demonstrated that erythritol has a significant impact on gene expression in the fly, potentially leading to impaired survival, motility, and egg laying. Key genes potentially affected by erythritol are listed in Table 1.

### 2.3. GO Enrichment Results of Differentially Expressed Genes

**Figure 3:**
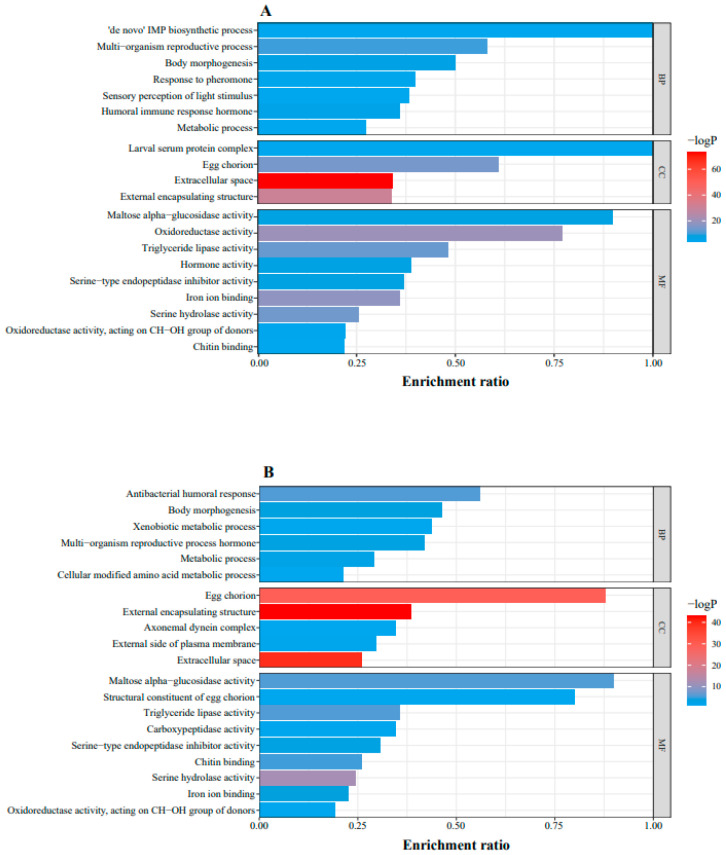
(**A**) GO enrichment results between erythritol-treated flies and control flies after 72 h. (**B**) GO enrichment results between erythritol-treated flies and control flies after 96 h. BP: biological process, DEGs: differentially expressed genes, CC: cellular component, MF: molecular function, enrichment ratio: the ratio of DEGs enriched into GO term to all genes in GO term.

GO enrichment results were ranked according to their *p*-values from smallest to largest. The top 20 GO enrichment results were performed using Metascape (Figure 3). Entries for body morphogenesis, humoral immune response, xenobiotic metabolism, hormone metabolism, and pheromone response were enriched in the biological processes (BPs), suggesting that erythritol induced a series of immune responses and affected fly growth. Entries associated with extracellular space and eggshell production were grouped into cell components (CCs). Oxidoreductase activity and hormone activity enriched in the molecule function (MF) category may be related to the immune response in the BP. Triglyceride lipase activity and maltase/α-glucosidase exhibited 45% and 90% of DEGs, indicating the abnormal energy metabolism of the fly. Egg chorion exhibited 60% of DEGs and all of the genes in the egg coat showed significant differential expression, indicating that the egg formation of the fly was impeded.

### 2.4. Enrichment Results of KEGG Pathways for Differentially Expressed Genes

**Figure 4:**
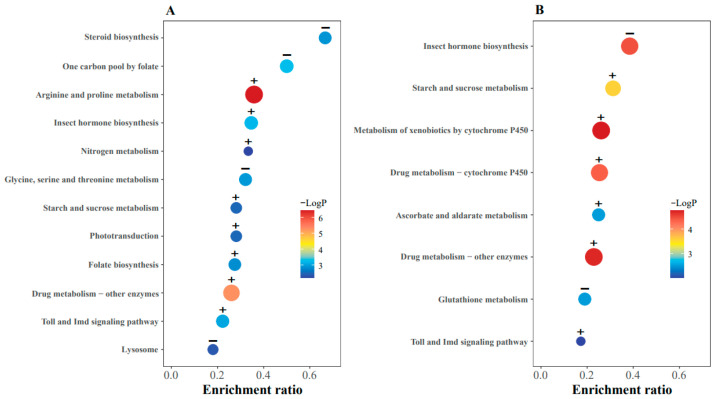
(**A**) KEGG pathway enrichment results between erythritol-treated flies and control flies after 72 h. (**B**) KEGG pathway enrichment results between erythritol-treated flies and control flies after 96 h, DEGs: differentially expressed genes, enrichment ratio: the ratio of DEGs enriched into KEGG pathways to all genes in KEGG pathways, +: up-regulated pathway, −: down-regulated pathway.

As shown in Figure 4, enrichment of the Toll and Imd signaling pathway, drug metabolism, and xenobiotics metabolism pathway imply that erythritol is recognized as an exogenous substance, subsequently triggering a series of defense responses in the fly. Nine genes (*Amyrel*, *Mal-A1*, *Mal-A2*, *Mal-A3*, *Mal-A4*, *Mal-A7*, *Mal-A8*, *Mal-B1* and *Mal-B2*) associated with the starch and sucrose metabolism pathway were highly up-regulated in flies fed with erythritol. Genes related to the insect hormone pathway, folate biosynthesis, and amino acid metabolic processes were also enriched.

### 2.5. Key Genes Associated with Carbohydrate Metabolism

**Figure 5:**
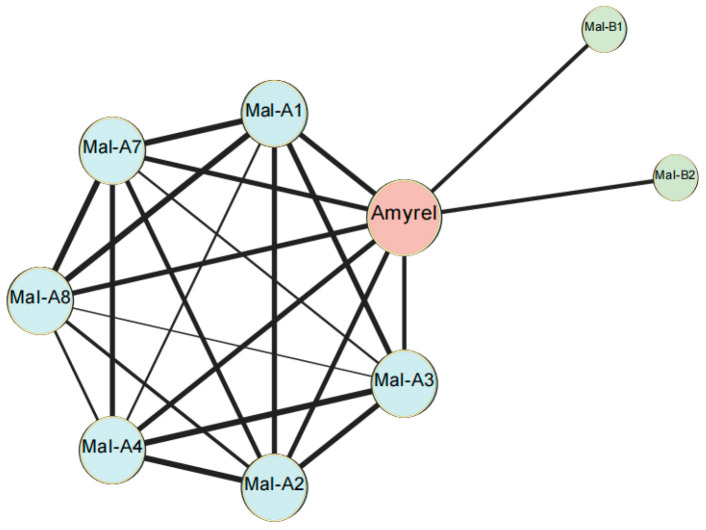
Interactions of key enzymes associated with starch and sucrose metabolism. The amylase gene Amyrel is linked to all other maltase genes in the pathway and is a hub gene of the pathway. *Mal-A1*, *Mal-A2*, *Mal-A3*, *Mal-A4*, *Mal-A7*, and *Mal-A8* are all linked to each other.

Genes associated with carbohydrate metabolism were further analyzed to explore the effects of erythritol on fly mortality. The differentially expressed gene *Amyrel* was significantly up-regulated in flies exposed to erythritol. *Amyrel* possesses both hydrolytic α-amylase and a 4-α-glucosyltransferase transglycosylation activity that hydrolyzes starch and related polysaccharides into maltose and maltotriose. It is noteworthy that eight DEGs encoding maltase genes (*Mal-A1*, *Mal-A2*, *Mal-A3*, *Mal-A4*, *Mal-A7*, *Mal-A8*, *Mal-B1* and *Mal-B2*) were found. Maltase genes hydrolyze glucose and maltose to produce energy. Up-regulated gene expression and activation of related metabolic pathways indicated an abnormal energy state in flies fed with erythritol. PPI (protein–protein interaction) network of maltase genes is shown in Figure 5.

### 2.6. Tissue-Specific Enrichment Results of Key Genes

**Figure 6:**
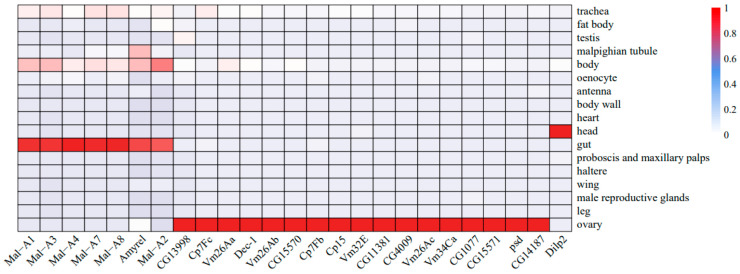
Heatmap of RNA-Seq data deconvolution results. The expression values of the genes in the 17 tissues were normalized to a range of 1 to 0, with 1 indicating the largest expression value. *Mal-A1*, *Mal-A2*, *Mal-A3*, *Mal-A4*, *Mal-A7*, *Mal-A8*, and *Amyrel* are highly expressed in the gut. *Dilp2* is highly expressed in the head. Other genes associated with egg laying are highly expressed in the ovary.

To understand the roles of key genes involved in mortality and sterility, their tissue-specific enrichment results were predicted (Figure 6). In statistical results from the Fly Cell Atlas data and deconvolution results of RNA-Seq data, the amylase gene (*Amyrel*) and six maltase genes (*Mal-A1*, *Mal-A2*, *Mal-A3*, *Mal-A4*, *Mal-A7*, and *Mal-A8*) were highly expressed in the gut. Seventeen genes associated with eggshell assembly (*Dec-1*, *Vm26Aa*, *Vm26Ab*, *Vm26Ac*, *Vm34Ca*, *psd*, *Cp7Fb*, *Cp7Fc*, *Cp15*, *Vm32E*, *CG11381*, *CG15571*, *CG15570*, *CG1077*, *CG4009*, *CG14187*, and *CG13998*) were highly expressed in the ovary. The expression of insulin-like peptide gene *Dilp2* was significantly increased in the head.

### 2.7. qRT-PCR Validation

**Figure 7:**
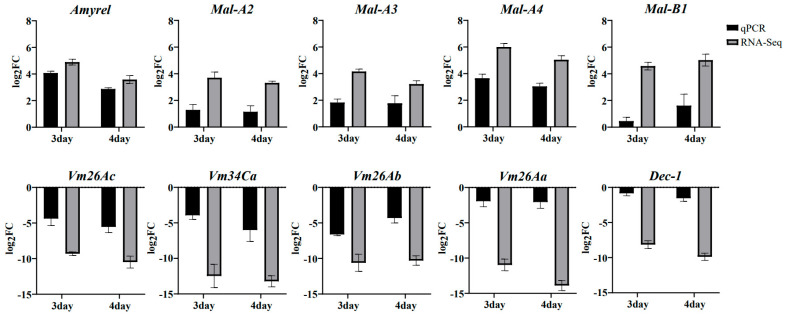
Comparison between qRT-PCR and transcriptome results of key genes. The expression profiles of all 10 selected genes showed the same trend as the transcriptomics sequencing results.

To validate the reliability of DEGs identified in transcriptome analysis, the expression profiles of ten key genes associated with starch metabolism (*Amyrel*, *Mal-A2*, *Mal-A3*, *Mal-A4*, and *Mal-B1*) or egg formation (*Vm26Ac*, *Vm26Ab*, *Vm26Aa*, *Vm34Ca*, *Dec-1*) were determined using qRT-PCR. Our results showed that gene expression levels in the RNA-seq data were significantly correlated with their expression detected by qRT-PCR (Figure 7).

## 3. Discussion

To gain a deeper insight into the molecular mechanisms underlying the impact of erythritol on fly mortality and oviposition, transcriptome sequencing was conducted on flies fed with or without erythritol. Subsequently, key genes were identified through RNA-Seq and bioinformatic analysis. Our results showed that an amylase gene and eight maltase genes were significantly upregulated in flies fed with erythritol medium. In addition, expressions of seventeen genes associated with egg-shell assembly were dramatically suppressed. It is notable that eleven genes proven to be involved in the regulation of hemolymph osmolality were not affected by erythritol.

### 3.1. Variation of Hemolymph Osmolality Is Not Necessary for Fly Death

Our findings indicate that the lethality observed in flies was not attributed to an osmotically driven effect. Several rational explanations support this conclusion: (1) flies possess a robust osmoregulation ability, allowing them to adjust osmotic balance by modulating the hemolymph volume during water deficit. Even when the hemolymph volume was reduced to 25% or less, osmolality remained relatively stable during a short dehydration period [19]. (2) Mortality resulting from significant osmolality variation may not occur, as the observed osmolality changes were well within the fly’s regulatory capacity. (3) The expression levels of three diuretic hormone genes (*DH44*, *DH31*, and *CAPA*) [20,21] and eight aquaporin genes (*Drip*, *Big brain*, *AQP*, *Eglp2*, *Eglp3*, *Prip*, *Eglp4*, and *Eglp1*) [22] did not differ significantly between the treatment and control groups. In conclusion, the osmolality variations induced by erythritol fall within the fly’s regulatory capacity and are not the primary cause of mortality.

### 3.2. Energy Deficiency Caused by Erythritol

**Figure 8:**
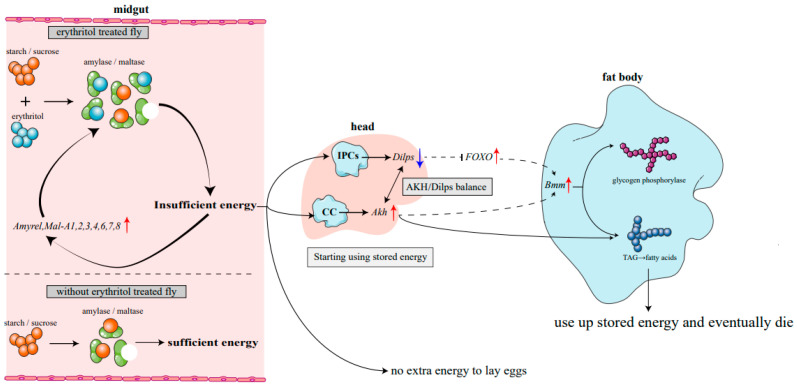
Molecular mechanism of effects of erythritol feeding on flies. IPCs: insulin-producing cells, CC: corpora cardiaca. Erythritol consumption induces energy deficiency in flies, prompting the utilization of stored energy. Flies consistently experience energy deficiency, with no surplus energy for reproduction, ultimately leading to mortality upon depletion of stored energy. 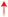: up-regulated genes. 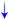: down-regulated genes.

α-glucosidases (EC 3.2.1.20) hydrolyze α-1,4-glycosidic bonds of starch, producing many maltose molecules and then digested by maltases into α-D-glucose [23]. A previous study showed that maltase genes (*Mal-A2*, *Mal-A3*, and *Mal-A4*) were responsible for dietary carbohydrate changes and could increase the response capacities associated with environmental variations [24]. As a competitive inhibitor, erythritol competitively binds α-glucosidase with the substrates starch or maltose, resulting in ineffective hydrolysis of substrates. In this scenario, even if the food is sufficient, it still leads to a low-energy state. The low-energy signal induces the production of more hydrolytic enzymes attempting to obtain sufficient energy, thus instigating a vicious cycle (Figure 8).

### 3.3. Mortality Caused by Energy Deficiency

Studies showed that there are two lipolytic systems in the insect fat body: *Akh*/*AkhR* signaling-dependent lipolysis [25] and *Bmm*-related lipolysis [26]. *Akh* is produced by corpora cardiaca cells in the head and stored within secretory vacuoles until signaled to release. Under acute starvation or increased metabolic demand, secretion of *Akh* into the hemolymph stimulates lipolysis of triacylglycerols, and conversion of glycogen into trehalose by *Akh* receptor (*AkhR*) [27] and consequently activating cAMP/PKA signaling pathway in the fat body [28]. As the ortholog of mammalian adipose triglyceride lipase (ATGL), *Bmm* catalyzes the initial step of triacylglycerol to diacylglycerol [26]. It must be noted that the lipolytic events mediated by TGL and *Bmm* are activated at different times by different metabolic signals. Furthermore, the *Akh* system functions in response to rapid changes in lipid demands, whereas *Bmm* functions to maintain lipid levels for the metabolic baseline [28].

The fly genome contains seven genes coding insulin-like peptides (*Dilp1-7*), which are homologous to the mammalian insulin and insulin-like genes. Secreted *Dilps* bind to the insulin-like receptor (*InR*) in the target tissues, activating the downstream components sequentially, including Pi3K92E, AKT1, mTOR and FOXO. Finally, activated insulin signaling exerts its effect on growth, development, metabolism, behavior, life span and immunity [29,30,31]. However, the secretion of *Dilps* depends on the developmental stage, type of tissue, and environmental factors [32]. *Dilp2*, *3* and *5* are produced in a cluster and are believed to be particularly important in the regulation of metabolism in the fly. *Dilp3* is specifically dedicated to the systemic control of circulating sugars, while *Dilp2* responds to amino acid metabolism [33].

A rational mechanism has been proposed to explain the relationship between *Dilps* and *Akh* in the fly. *Dilps* and *Akh* are two counter-regulatory molecules that regulate glucose level in the fat body [34]. Starvation (or energy deficiency) can induce the expression of *Akh*, promoting glycolysis. In addition, *Akh* can regulate the expression of *Bmm*, which catabolizes lipids and maintains the energy balance. Therefore, the balance between *Dilps* and *Akh* may be important for resource allocation into growth and reproduction. In this study, competitive binding of erythritol and maltase to sucrose and/or maltose significantly inhibited their hydrolysis, resulting in energy deprivation. The energy deprivation signal triggered higher expression of *FOXO*, *Akh* and *Bmm* but suppressed the expression of *Dilp2*, *Dilp3* and *Dilp5*, indicating that stored lipids and glycogen were overused. The combined effects of the depletion of stored lipids and the failure of dietary sugars to provide sufficient energy accelerated the mortality of the fly (Figure 8).

### 3.4. Molecular Mechanism of Non-Oviposition Caused by Erythritol

In the fly, the *defective chorion-1* gene (*Dec-1*) encodes follicular cell proteins required for normal eggshell assembly. *Dec-1* encodes fc177 (177 kDa), fc125 (125 kDa), and fc106 (160 kDa) via selective splicing of RNA [35]. They are secreted by follicular cells and localized in the yolk membrane layer, where they are further cleaved into at least five different proteins [35,36]. Ultrastructural analysis of the *Dec-1* mutant eggshell revealed abnormalities in the endochorionic layer and endosperm [37]. The aggregation of chorionic material beneath the vitelline membrane caused eggshell malformation. In this study, *Dec-1* expression was reduced by approximately 296-fold in flies fed with erythritol, suggesting that erythritol may cause female sterility by affecting eggshell assembly. Previous studies have indicated that *Vm26Ab*, *Vm26Ac*, *Vm26Aa*, *Vm34Ca*, *Vm32E*, and *Vml* are essential for vitelline membrane formation. The high expression of *Vm26Aa*, *Vm26Ab*, *Vm26Ac*, *Vm34Ca*, *Vm32E*, and *Vml* in mated females significantly promotes oviposition [38]. Sv23, encoded by *VM26Ab*, is expressed in follicle cells and yolk membranes, playing a role in the formation of yolk membrane and chorion-containing eggshell [39]. Knockdown experiment targeting *Vm26Ab* has shown a significant reduction in oocyte numbers [38]. Chorionic protein is synthesized by follicular cells and secreted into the extracellular space between the follicle cells and the developing oocyte. There are several chorionic membrane protein families in the fly, and each family is expressed at a specific stage of oogenesis [40]. Protein products of *Cp7Fb*, *Cp7Fc*, *Cp7Fa*, *Cp36* and *Cp15* are essential for a normal chorionic membrane lining structure, and their deficiencies result in chorionic membrane fragility. *Cp7Fa*, *Cp7Fb* and *Cp7Fc* were located in an amplified chorionic gene cluster including *Cp36* and *Cp38* [41]. *Cp36* was proven to produce a major structural protein of the chorionic membrane. It is synthesized in the early stages of oogenesis and initially deposited in the vitelline membrane but later concentrated in the chorionic villous layer. Mutations in *Cp36* resulted in defective cross-linking of vitreous and chorionic proteins [42]. A study revealed that female sterile *fs(1)M3* could be accumulated in the vitelline membrane together with the female sterile *fs(1)N* and plays a role in vitelline integrity and activation of Torso receptors [43]. Palisade (*Psd*) is required for the assembly and function of the protective vitelline membrane in the fly ovary. After the knockdown of *Psd* by RNAi, the somatic cells surrounding the oocyte showed structural disorders during the initial synthesis of the vitelline membrane, including extensive size differences between precursor vitelline bodies and disorganization of follicular cell microvilli [44]. The *CG11381* gene encodes a glutamine-rich protein with an expression pattern that temporally bridges with the major follicular membrane and early intermediate chorionic membrane proteins [45]. In this study, non-oviposition was caused by significant down-regulation or non-expression of genes encoding vitelline membrane proteins and chorionic membrane proteins in flies fed with erythritol. The fly prefers to survive rather than reproduce in the absence of energy.

## 4. Materials and Methods

### 4.1. Fly Rearing and Treatment

The fly rearing normal medium consisted of the following components: sucrose (62 g), corn powder (82 g), agar (6.2 g), dry yeast (7 g), and propanoic acid (2 mL), and it was fixed to 1000 mL with distilled water. The erythritol medium contained an extra 122 g (1 mol/L) of erythritol. Newly emerged flies were transferred to culture flasks, with each flask containing 30 flies.

A total of 120 flasks (3600 flies) were collected, with half designated for the experimental group fed with erythritol medium and the other half for the control group fed with normal medium. These flasks were then placed in an incubator at a temperature of 25 ± 1 °C, under a photoperiod of 12 h of light followed by 12 h of darkness. The relative humidity was maintained at 70 ± 5%. Daily fly survival numbers were counted, and survival analysis was performed using the Kaplan–Meier method using the survival R package version 3.5-7.

### 4.2. RNA Extraction, Library Construction and Sequencing

One hundred flies were collected on the third or fourth day with three biological replicates after being fed with or without erythritol for RNA extraction. Total RNA was extracted with the Trizol reagent and quantified using an Agilent 2100 bioanalyzer. RNA sequencing libraries were constructed using the NEBNext^®^ Ultra™ RNA Library Prep Kit for Illumina^®^ (New England Biolabs, Ipswich, MA, USA). mRNAs were enriched using Oligo(dT) magnetic beads, followed by random fragmentation in NEB Fragmentation Buffer. The first strand of cDNA was synthesized in the M-MuLV reverse transcriptase system using fragmented mRNA as templates and random oligonucleotides as primers. After RNA removal with RnaseH, the second strand of cDNA was synthesized using DNA polymerase I. Purified dscDNA was end-repaired, an adapter was added, the PCR was amplified, and then sequencing was carried out on the Illumina NovaSeq 6000 sequencing platform (Novogene Bioinformatics Technology Co., Ltd., Beijing, China). The data can be downloaded on GEO (accession number: GSE221267).

### 4.3. Data Quality Control and Reference Genome Alignment

To ensure data quality and reliability, sequencing adapters, N (unidentifiable base), and low-quality reads (Qphred ≤ 20) were removed. The resulting clean reads were then aligned to the reference genome (dmel_r6.37_FB2020_06) using HISAT2 version 2.2.0.

### 4.4. Bioinformatic Analysis

#### 4.4.1. Identification of Differentially Expressed Genes and Enrichment of GO and KEGG

Differentially expressed genes (DEGs) were identified using the Deseq2 R package version 1.40.2 in R based on count data [46]. The screening criteria were Padj < 0.05 and |log2FC| ≥ 1.5. GO and KEGG pathway enrichment were performed using the online website Metascape [47] with the following parameters: min overlap = 3, *p*-value cutoff ≤ 0.01 and min enrichment = 1.5. To gain insight into the state of a pathway (activation or suppression), enriched KEGG pathways were scored using the GSVA R package version 1.50.1 [48] with the following equation:

GSVA enrichment score = (X − Y)/Y

where X and Y are the GSVA scores for the treatment group and the control group, respectively.

#### 4.4.2. Hub Genes Screening of KEGG Pathways

The PPI network of DEGs in enriched KEGG pathways was predicted using the STRING tool version 11.5 [49] with the threshold of a combined score > 0.4. Cytoscape version 3.10.1 [50] plug-in CentiScaPe version 2.2 [51] was then used to assess the centrality of DEGs in the PPI network with three parameters: degree, betweenness and closeness. DEGs with 3 parameters larger than reference values were defined as hub genes in this study.

#### 4.4.3. Tissue-Specific Enrichment Analysis of Key Gene

To explore gene expression profiles in the specific-tissue type, the BayesPrism algorithm was used to deconvolve bulk RNA-Seq data using scRNA-Seq data from 17 tissues as references. scRNA-Seq data of 17 tissues of the fly were downloaded from FLY CELL ATLAS [52], then gene expression matrices were extracted from scRNA-Seq data using the *Connect* function in the SeuratDisk [53] R package. These 17 matrices were then integrated and metadata for all cells were generated. The *new.prism* function in the BayesPrism version 2.1.1 [54] R package was used to create a prism object using the integrated scRNA-Seq data, bulk RNA-Seq data and metadata as inputs. The parameter settings were as follows: key = null, outlier.cut = 0.01, outlier.fraction = 0.1. Finally, the *run.prism* function was used with the created prism object as an input to deconvolve the bulk RNA-Seq data and generate gene expression profiles for the 17 tissues.

### 4.5. Validation of the DEGs by Quantitative Real-Time PCR (qRT-PCR)

To validate the RNA-seq results, 10 DEGs involved in fly death or egg formation were selected for qPCR analysis. Sequence-specific primer sets were designed using Primer Premier 6 and are listed in Supplementary Material S1. Total RNA was extracted using AG RNAex Pro RNA Extraction Reagent (Accurate Biology, Changsha, China) according to the manufacturer’s instructions. cDNA was synthesized using the TransScript^®^ All-in-One First-Strand cDNA Synthesis SuperMix for qPCR (TransGen, Beijing, China) according to the manufacturer’s instructions. Each 50 μL qPCR reaction mixture contained 2.5 μg RNA, 2.5 μL gDNA Remover, 10 μL 5× SuperMix, 35 μL ddH_2_O. PCR reactions were performed using the BIO-RAD CFX96 system with the following parameters: 95 °C for 120 s, followed by 40 cycles of 95 °C 10 s, 60 °C 15 s, and 72 °C 15 s. The Forkhead box K (*FoxK*) was used as an internal reference gene [55]. The expression of each gene was conducted in three biological replicates and three technical replicates. Relative transcription levels of target genes were determined by employing the 2^−ΔΔCt^ method [56].

## 5. Conclusions

In this study, RNA-seq and bioinformatic analysis were performed to clarify that the cause of erythritol-induced death in the fly was energy depletion as opposed to a drastic change in hemolymph osmolality. Energy depletion severely affected energy allocation. Flies that ingested erythritol had to save energy for survival, but not for reproduction.

## Figures and Tables

**Table 1:** Detailed information of key differentially expressed genes.

Gene	Enriched Tissue	Category	Functions	Log2FC
*Dilp2*	head	insulin-like peptide	carbohydrates/lipids metabolism	−2.4↓
*Dilp3*				−1.8↓
*Dilp5*				−2.6↓
*Akh*	body	adipokinetic hormone	carbohydrates/lipids metabolism	1.6↑
*Mal-B1*	trachea	α-glucosidase	carbohydrate metabolism	4.6↑
*Mal-B2*	fat body	α-glucosidase	carbohydrate metabolism	2.1↑
*Bmm*		triglyceride lipase	lipid/triglyceride metabolism	2.8↑
*Amyrel*	gut	α-amylase	carbohydrate metabolism	4.9↑
*Mal-A1*		α-glucosidase		3.0↑
*Mal-A2*				3.7↑
*Mal-A3*				4.3↑
*Mal-A4*				6.0↑
*Mal-A6*				2.7↑
*Mal-A7*				2.9↑
*Mal-A8*				2.3↑
*Dec-1*	ovary	defective chorion	eggshell assembly	−8.21↓
*Vm26Ab*		vitelline membrane	major early eggshell protein	−10.7↓
*Vm26Ac*				−9.3↓
*Vm26Aa*				−11.0↓
*Vm34Ca*				−12.3↓
*Vm32E*				−12.2↓
*Psd*			act with the vitelline of eggshell	−9.0↓
*Cp7Fb*		chorion protein	egg protection	−6.5↓
*Cp7Fc*				−6.6↓
*Cp15*				−4.9↓
*Cp36*				−4.0↓
*fs(1)M3*				−2.3↓
*fs(1)N*				−2.2↓
*CG1077*			chorion containing eggshell formation	−5.0↓
*CG4009*				−5.5↓
*CG14187*				−6.4↓
*CG13998*				−5.5↓
*CG11381*			egg chorion	−6.1↓
*CG15571*				−2.5↓
*CG15570*				−5.4↓

## Data Availability

RNA sequencing count files were deposited in the NCBI Gene Expression Omnibus (GEO; Accession GSE221267).

## Supplementary Materials

The following supporting information can be downloaded at: https://www.mdpi.com/article/10.3390/ijms25073738/s1.
