# Supplementary material for: Comparative Transcriptomic Analysis Revealing the Potential Mechanisms of Erythritol-Caused Mortality and Oviposition Inhibition in Drosophila melanogaster
Source: Int J Mol Sci. 2024 Mar 27;25(7):3738. doi: 10.3390/ijms25073738 (PMC11011834; doi:10.3390/ijms25073738)
Supplement: Supplementary file 1 [file ijms-25-03738-s001.zip › ijms-2901923-supplementary.pdf]

Gene name	Forward	Reverse
FoxK	GTTGGTGGTGGTGGTGGAGTA	GTGGTGTGGATGCTGCTGTTG
Amyrel	GCTGGTGGGACAATGGAGACA	CGGCAGGCAGACAGGTATTCA
Dec	TCGCAAGAGGCACAGATGAAGA	GGCATTCGGAACCAGAGGAGA
Mal-A2	GGAGGCGATTCACGGGTTCT	ACATCCTTGGCGGTGGAGTATC
Mal-A3	GGCAAGGTGGTCAACGGAATC	GGCGTGGAATTGGTGGAGGTA
Mal-A4	CGCAACGATTGGACCAACGAT	GCTCATCACCGCCATTGTCAG
Mal-B1	TCGCAGGGTCTTCGCTTTCA	CGCCACAAGGACACTCAATCG
Psd	CGGTGGATGTGGTCAGAACAAC	CTACATAGCCTGCATCGCCATC
Vm26Aa	CCTTCGTGTGCATCGCTCTG	AGGTCACCGAATCCCTGTCC
Vm26Ab	TGGCATTCAACTTTGGTCACCT	GCGACACACGGATGTTCTCG
Vm26Ac	CGCAGCTACGACCAGGATGA	CTTGATAACCGCTTCGCAGGAG
Vm26Ca	AGTGCATCGCCATCGTCTCC	TTGCCGTAGCCACCACCGTA
